# Ensuring Global Health Equity in a Post-pandemic Economy

**DOI:** 10.34172/ijhpm.2022.7212

**Published:** 2022-06-14

**Authors:** Ronald Labonté

**Affiliations:** Globalization and Health Equity, School of Epidemiology and Public Health, Faculty of Medicine, University of Ottawa, Ottawa, ON, Canada.

**Keywords:** Post-Pandemic Economy, Health Equity, Green Recovery, Degrowth

## Abstract

With coronavirus disease 2019 (COVID-19) receding, many countries are pondering what a post-pandemic economy should look like. Some advocate a more inclusive stakeholder model of capitalism. Others caution that this would be insufficient to deal with our pre-pandemic crises of income inequality and climate change. Many countries emphasize a ‘green recovery’ with improved funding for health and social protection. Progressive tax reform and fiscal policy innovations are needed, but there is concern that the world is already tilting towards a new round of austerity. Fundamentally, the capitalist growth economy rests on levels of material consumption that are unsustainable and inequitable. More radical proposals thus urge ‘degrowth’ policies to reduce consumption levels while redistributing wealth and income to allow the poorer half of humanity to achieve an ethical life expectancy. We have the policy tools to do so. We need an activist public health movement to ensure there is sufficient political will to adopt them

 As the world enters a third pandemic year, we seem close to a return to ‘normalcy.’ After a year of rich country vaccine-hoarding, half of the world’s population is expected to have access to a sufficient supply of vaccines by mid-2022 and quantity is no longer the major concern in reaching global vaccination targets. But the “road to recovery has never been a particularly smooth one.”^1^ And despite many billions eager to end the disruptions severe acute respiratory syndrome coronavirus 2 (SARS-CoV-2) has imposed on their lives and livelihoods should we be eager to return to the ‘normal’ we left behind in early 2020? If the health of people and planet are of any concern, the answer is a resounding no. Prior to the pandemic, surging disparities in wealth and power undermined health gains, climate crises threatened human survival, the mass migrations of people were increasingly met by xenophobic populism, and the slow-burn rise in antimicrobial resistance was causing more deaths annually than HIV or malaria.^2^

 That a post-COVID-19 (coronavirus disease 2019) economy should look different was already mooted in the early months of the pandemic:

- The World Economic Forum (WEF) called for a ‘Great Reset’ to ‘stakeholder capitalism.’
- The United States and other countries promised to ‘Build Back Better.’
- The European Union (EU) and most other countries committed to a ‘green recovery.’

Others called on governments to regulate markets to ensure economic activities achieved democratically determined health, social, and environmental goals. Some called for more radical proposals to transition rapidly to a ‘degrowth’ or ‘post-growth’ economy in which the world went on an extreme diet of material consumption. Common to all proposals was agreement on the common denominator linking diverse groups whose health suffered most from COVID-19: socioeconomic inequality:

*“The pandemic brought home to us a hard truth. Unequal access to incomes and opportunities does more than create unjust, unhealthy and unhappy societies—it kills people*.”^3^

 Underpinning socioeconomic inequality are economic policies and practices that have allowed a tiny sliver of humanity – the world’s billionaire class – to become wealthier as the overwhelming majority became poorer.^4^ In this disequalizing context the question remains: what sort of post-pandemic economic world should we strive to achieve, if health equity and environmental sustainability are to be our collective goals?

## From Shareholder to Stakeholder Capitalism: More of the Same?

 The political economy that characterizes most of today’s world – capitalism – is centuries old, although its most recent form – neoliberalism – only became dominant in the 1980s.^2^ Neoliberalism’s core elements (trade and financial liberalization, low taxation, minimal state intervention, strong property rights) birthed our now familiar globalized economy, criticized almost at its outset for the inequalities it was fomenting. To WEF founder, Klaus Schwab, the problem lay less with capitalism itself than with its neoliberal emphasis on maximizing shareholder value, where economic decisions are based on creating the greatest return on investment in the shortest time possible. Schwab’s and the WEF’s promoted solution is ‘stakeholder capitalism,’ in which everyone (and not just shareholders) should have a stake in capitalism’s benefits.^5^ Few might object to the idea of all gaining (even if not equally) from liberalized market activities, but as one economist argued, the stakeholder model is essentially:

*“…just a way of bringing the opponents of capitalism to a common venture of extending its lifespan, while ignoring the system’s intrinsic and destabilizing profit motive” *(W. Bello, interview communication, October 27, 2021).

 As one example, in a post-pandemic ‘Great Reset’ wealthy investors are encouraged to invest in businesses whose activities align with the Sustainable Development Goals. This, it is said, would allow them to “make a profit and still save the world.”^6^ To the extent that such investments go into non-fossil-fuel renewables this win-win hyperbole is a partial truth; but with many of these ethical investment portfolios being non-compliant with global climate change goals^7^ this may simply be ‘greenwashing’ what remains fundamentally profit-motivated investing. We might also ask: What economic policies and practices enable investors to become disproportionately wealthy in the first place? As a recent study noted, the stakeholder capitalism model will do little to redistribute wealth but will strengthen the private sector’s (not unself-interested and growing) role in global health governance.^8^

## The Return of the State: Can Governments Mitigate Capitalism’s Inherent Inegalitarianism?

 The state is certainly a critical ‘stakeholder’ in capitalism since its policy choices enable or constrain the actions of economic actors within and across borders. The post-1980 drift to neoliberalism saw the state increasingly defer to market interests in efforts to have their countries remain globally competitive (T. Jackson, interview communication, October 28, 2021).^9^ Tax rates fell, financial markets deregulated, and inequalities within most countries soared.^2^ The 2008 global financial crisis saw a rapid *volte-face*, with wealthier country governments spending trillions of dollars to bail out ‘banks too big to fail.’^2^ The return of the state was brief, with austerity measures (fiscal contraction) quickly following to cover the public costs of rescuing private banks and investors^2^ at the cost of eroding health systems that proved ill-prepared for a global pandemic.

 The pandemic saw the state roar back once again, with many countries responding with wage support, cash transfer, credit schemes, tax cuts and delays, support to importers and exporters, policy rate cuts, support to businesses, and rent subsidies or deferrals.^10^ This fuelled speculation of a turning point in state/market dynamics, which some attributed to the COVID-19 crisis being different from the one in 2008:

*“It showed us that the people who matter most in society, the ones who protect our lives and care for us, the ones we applauded from our doorsteps during the pandemic, yet they were…left behind in terms of wages, security, the value of their jobs, their status in society” *(T. Jackson, interview communication, October 28, 2017).

 Certainly, the need for greater public investment in health and social protection is an undisputed outcome of the pandemic, especially given how government responses to COVID-19 increased women’s care burdens, employment losses and experiences of domestic violence.^10-12^ Others saw massive government interventions in the economy as demonstrating that:

*“…all that neoliberal talk about government intervention is bad, bad, bad just got thrown out the window…what we’ve seen is the ideological assumption of neoliberalism laid low” *(W. Bello, interview communication, October 27, 2021).

 Early post-pandemic recovery plans for those countries with the requisite fiscal capacities appeared to embody such transformative optimism. The original US multi-trillion Green New Deal promised substantial environmental protection, a rapid shift away from fossil fuels, and expansive new social spending.^13^ When it was later tabled as a ‘Build Back Better’ plan its ambitions were trimmed substantially, and then even more due to fossil fuel industry lobbying and the rise in right-wing populism.^14^ There are concerns that polarized politics in the USA may prevent it from ever being enacted.^15^ The EU’s ‘Next Generation’ plan^16^ has similar intentions to be green and socially more just and, while insufficiently generous,^17^ it fares better than its American counterpart. It could still be undone by the EU’s own right-wing drifts and, with geopolitical tensions rising globally, notably following Russia’s invasion of Ukraine, the EU and many other countries are pivoting back to energy (fossil fuel) independence, questioning their 2021 commitments to achieve net-zero emissions by 2050.^18^

## Tax and Fiscal Policy Space: Can We Build Back Fairer?

 The potential for a substantially reformed and more state-engaged capitalism still exists. At a minimum the tax roll-backs that Organisation for Economic Co-operation and Development (OECD) countries began embracing in the 1980s need a rapid reversal. Marginal income, dividend, corporate, wealth, and inheritance tax rates can all be increased substantially without negatively affecting (more likely improving) the quality of life for most.^19^ This should be easiest for high-income countries which hold most of the world’s economic wealth; it will be more challenging but not impossible for low-income countries with large informal labour markets and low gross domestic product/capita. There is also a need to reimpose or strengthen border control measures to stop capital flows from cash-strapped poor countries to tax havens, a corporate practice (often illicit) that costs developing countries hundreds of billions of dollars annually.^20^

 Governments for some years have recognized the need to develop international taxation systems fit for a globalized economy. The 2021 agreement by 136 countries to implement a minimum 15% tax rate for multinational enterprises is a start, albeit at too low a rate to have much impact and with tax benefits likely to benefit wealthy countries disproportionately.^21^ A small financial transaction tax applied internationally on currency exchange could create trillions more in shareable public revenues.^21^ To put the global tax justice issue into some perspective: in 2002 the total untaxed monetized value of the global economy was $29.8 trillion. In 2019, this had swelled to $74.5 trillion.^21^ There is no shortage of wealth, only a paucity in the fairness of its allocation and the health and social benefits (and pandemic preparedness) this would create.

 The global financial and COVID-19 crises both saw some governments adopt unusual fiscal responses (issuing bonds held by their central banks) to generate trillions in new money used to bail-out financial institutions, stimulate domestic economies, or provide pandemic relief. Described as ‘modern monetary theory’ (MMT), adherents argue that states with their own sovereign currency can never run out of money.

*“[And] that fundamental insight gives us the space that we need to create monetary and fiscal policies that are flexible, that are coordinated and that give government the space to maneuver as we navigate these huge environmental and social challenges that are facing us…lifting the veil of the ideology that says the government cannot afford to spend in the well-being of its citizens” *(T. Jackson, interview communication, October 28, 2017).

 MMT was invoked by the World Health Organization’s (WHO’s) Council on the Economics of Health for All, established in November 2020 with the aim of ensuring “that national and global economies are structured…to deliver on this ambitious goal.”^22^ The Council’s first policy brief chided governments for failing to impose conditions on the public monies that funded COVID-19 vaccines that would have required companies to share their technology^23^ rather than allow monopoly intellectual property rights to create ‘vaccine apartheid’ and pharmaceutical profiteering.^24^ Its second brief noted how a combination of progressive tax and fiscal measures (including MMT) could ensure that health and social protection systems are sufficiently strong to mitigate any future pandemic or other health crisis.^25^

 There are, however, two problems with MMT as currently practiced. First, “central-bank resources (balance sheets) have been expanded and deployed in the private interests of vast, unregulated, and systemically risky capital markets across the ‘shadow banking’ system,”^26^ fuelling the speculative asset bubbles that saw billionaire wealth climb precipitously higher during the pandemic. Re-nationalizing much of these assets is important, as is reorienting central banks’ activities “away from…sustaining private gains in capital markets [and] toward public purpose.”^27^ Tax measures and price controls can be used to restrain any long-term risks of inflation,^28^ with some economists arguing that current inflation risks are being intentionally over-stated to justify a return to the same austerity measures that followed the 2008 crisis.^28-30^

 Second, not all countries have sovereign reserve currencies and must borrow on international markets, usually denominated in US dollars. This exposes them to volatile currency fluctuations and interest rate increases. Debt burdens (both public and private) are already extremely high, with many low- and middle-income countries on the verge of defaulting on their loans. Debt cancellation is one option, since some of this debt should be declared ‘odious’ and uncollectable.^31^ With fiscal consolidation already rising in these countries, there are calls for the International Monetary Fund (IMF) to issue Special Drawing Rights (SDRs), the Fund’s reserve currency, to support such countries’ pandemic recovery. SDRs are virtually interest-free and are non-conditional. The IMF already approved the release of US$ 650 billion in SDRs in response to the pandemic, but current rules mean that most of this amount is accessible only to high-income countries. Advocates are urging wealthier nations to allocate their share to low- and middle-income countries (some have) and for the IMF to issue an additional US$ 500 billion annually in SDRs for the next 20 years to finance climate change mitigation. The numbers are large, but still “trivial compared to the $25 trillion in liquidity fueled by loose monetary policies in advanced economies since the 2008 global financial crisis.”^31^

 Taxes, MMT, IMF reform: There are ways in which public wealth for public good purposes can be recaptured and equitably allocated. But doing so requires a shift in how states see their role in the economy, away from being the backstop to capitalism’s inevitable market failures to actively regulating (shaping) markets towards democratically determined health, social, and environmental goals.

## Degrowth/Post-growth: Should We Build Back at All?

 Two common elements in many post-pandemic plans are commitments to decouple economic growth from carbon intensity, and to embrace a ‘circular economy’ in which all goods are reused or repurposed to reduce material throughput and to eliminate (or massively reduce) waste. Such measures are essential, but many environmental economists are sceptical that they are sufficient. They also allow societies to avoid confronting ‘an inconvenient truth’^[1]^: That capitalism’s growth imperative is predicated on ever increasing levels of material consumption. The human population already consumes annually 1.7 times the ecological resources the world can regenerate. If everyone consumed at the level of OECD countries it would require the resources of 4.7 earths,^21^ even before accounting for the environmental damages such consumption generates.

 A relatively new concept has entered the lexicon of environmental economics: degrowth^[2]^, which captures the importance of reducing aggregate global consumption levels to avoid catastrophic ecosystem collapse. The bulk of this responsibility lies with citizens, governments, and corporate actors in the historically over-consuming Global North,^32^ partly to make space for those in the Global South to reach consumption levels compatible with healthy life expectancies while remaining within safe planetary limits:

*“…it’s abundantly clear it’s in the poorest parts of the world that income growth makes a huge difference to prosperity. Life expectancy increases. Infant mortality decreases. Maternal morbidity decreases. Participation in education increases…that’s where growth makes a difference”* (T. Jackson, interview communication, October 28, 2017).

 The implications of such a transition are enormous. The burden of change rests heaviest on the world’s wealthiest regardless of which country they may live in. Their carbon emissions and consumption levels massively exceed those of the majority of the world’s population, indicative of the link between rising economic inequality and environmental devastation.^33^ But a degrowth (or ‘fair growth’) economy will demand substantial change for hundreds of millions, and although:

*“…degrowth is the only alternative at this point in time, there will need to be political and social psychological transformations from societies that have been weaned on overconsumption. I will not underestimate this cost [since] we’re talking about transformations in the way that we have structured our lives*” (W. Bello, interview communication, October 27, 2021).

 Others describe this as transitioning to a post-growth economy, one in which the pursuit of ‘prosperity’ replaces that of growth:

*“Many of our problems, both social and structural and environmental, arise from the idea that progress is about increasing productivity, the speed with which we create material goods and services, distribute them to people to buy and throw away as fast as possible, the belief that this material sense of productivity and progress is what human prosperity is really about. But what we saw through the pandemic is that health is really the meaning of prosperity. Health is the foundation for prosperity*(T. Jackson, interview communication, October 28, 2017).

 This requires a very different economy:

*“…a care economy, one that enriches us as well as saving lives. It’s a lower carbon economy, with a lower footprint because it is about engagement and attention and time in the service of each other” *(T. Jackson, interview communication, October 28, 2017).

 Such an economy is an extension of what:

*“…feminist economists have always talked about. There is the direct care which is looking after people… Then there’s extended care…doing things that help in adaptation to climate change…it is essential work that is being done in unpaid fashion by a significant part of the population” *(J. Ghosh, interview communication, November 14, 2021).^[3]^

## Towards a Post-growth Caring Economy: Can We Challenge the Rise in Autocratic Regimes?

 Transforming from a consumption-based capitalism to a sustainable caring economy requires governments willing to discipline markets for public good purposes, and to initiate tax and fiscal policies that radically redistribute access to the resources people need for healthy lives. The immediate challenge to this aspirational goal is reversing the fifteen years’ worldwide decline in democratic accountability and parallel rise in authoritarian rule.^34^ One of the pandemic’s ironies is that even as it increased the state’s role in health and economic protection, it incentivized alt-right populism and provided opportunities for autocrats to increase their grip on power. As Walden Bello, a sociologist and economist who was interviewed for this editorial, noted:

*“We are in a race between the forces of the far right and progressive forces [which threaten] any sort of coordinated global action on climate” *(W. Bello, interview communication, October 27, 2021).

 In response, Bello ran as a vice-presidential candidate in the May 2022 Philippines elections in an effort to avoid that country’s “resurgence of authoritarianism.” He did not win, but remains committed to the scale of transition needed for a survivable post-pandemic economy, one in which democratic participation must remain strong:

*“We can’t leave it just to the politicians. We’ve got to have civil society stepping in because if we leave it to the usual actors, we’re not going to get anywhere*” (W. Bello, interview communication, October 27, 2021).

 Even as the space for such participation is under authoritarian attack, the importance of civil society efforts to retain and expand it is more important now than in the pre-pandemic period. We know the political economy tools that can bring us closer to what the Sustainable Development Goals describe as ‘the world we want.’ But only organized citizen demand will create the political will to adopt these tools, with recent history bringing us evidence of unwavering activism in global climate strikes, Black Lives Matter, *buen vivir*and peasant’s movements, and poor people’s campaigns worldwide. The post-pandemic public health imperative now is to protect and support such movements.^35^

## Acknowledgements

 This editorial draws from work undertaken for People’s Health Movement for the Prince Mahidol Awards Conference 2022. It includes comments from three economists interviewed as part of that work: Walden Bello, University of the Philippines Dilman; Tim Jackson, University of Surrey; and Jayati Ghosh, University of Massachusetts Amherst. A webcast of these interviews can be found here: https://www.youtube.com/watch?v=AnsmZdgcJaU.

## Ethical issues

 Not applicable.

## Competing interests

 Author declares that he has no competing interests.

## Author’s contribution

 RL is the single author of the paper.

## Endnotes

 [1] This is a reference to ‘An Inconvenient Truth,’ the title of the 2006 documentary on global warming narrated by form US presidential candidate, Al Gore. [2] Not all critics of the unsustainability of current material consumption levels like the term, degrowth, especially since consumption (and economic) growth for poorer persons and nations remain important in providing the means for people to achieve reasonable health and life expectancies. ‘Fair growth’ may be a more marketable concept, but that the wealthier deciles of the human population must ‘degrow’ their current consumption patterns remains to ensure sufficient consumption space for poorer populations to ‘grow’ (improve) their own health and wellbeing. [3] Some post-pandemic recovery plans include specific reference to the ‘care economy,’ and many include increased spending in health, social protection, childcare, and other social care sectors, including improved conditions for those employed in such work.
